# Hemodynamic and metabolic characteristics associated with development of a right ventricular outflow tract pressure gradient during upright exercise

**DOI:** 10.1371/journal.pone.0179053

**Published:** 2017-06-21

**Authors:** Annelieke C. M. J. van Riel, David M. Systrom, Rudolf K. F. Oliveira, Michael J. Landzberg, Barbara J. M. Mulder, Berto J. Bouma, Bradley A. Maron, Amil M. Shah, Aaron B. Waxman, Alexander R. Opotowsky

**Affiliations:** 1 Department of Cardiology, Academic Medical Center, Amsterdam, The Netherlands; 2 Netherlands Heart Institute, Utrecht, The Netherlands; 3 Pulmonary and Critical Care Medicine, Department of Medicine, Brigham and Women's Hospital and Harvard Medical School, Boston, Massachusetts, United States of America; 4 Division of Respiratory Diseases, Department of Medicine, Federal University of São Paulo (UNIFESP), SP, Brazil; 5 Department of Cardiology, Boston Children's Hospital, and Harvard Medical School, Boston, Massachusetts, United States of America; 6 Cardiovascular Medicine, Department of Medicine, Brigham and Women’s Hospital and Harvard Medical School, Boston, Massachusetts, United States of America; 7 Veterans Affairs Boston Healthcare System, Boston, Massachusetts, United States of America; Scuola Superiore Sant'Anna, ITALY

## Abstract

**Background:**

We recently reported a novel observation that many patients with equal resting supine right ventricular(RV) and pulmonary artery(PA) systolic pressures develop an RV outflow tract(RVOT) pressure gradient during upright exercise. The current work details the characteristics of patients who develop such an RVOT gradient.

**Methods:**

We studied 294 patients (59.7±15.5 years-old, 49% male) referred for clinical invasive cardiopulmonary exercise testing, who did not have a resting RVOT pressure gradient defined by the simultaneously measured peak-to-peak difference between RV and PA systolic pressures.

**Results:**

The magnitude of RVOT gradient did not correspond to clinical or hemodynamic findings suggestive of right heart failure; rather, higher gradients were associated with favorable exercise findings. The presence of a high peak RVOT gradient (90^th^ percentile, ≥33mmHg) was associated with male sex (70 vs. 46%, p = 0.01), younger age (43.6±17.7 vs. 61.8±13.9 years, p<0.001), lower peak right atrial pressure (5 [3–7] vs. 8 [4–12]mmHg, p<0.001), higher peak heart rate (159±19 vs. 124±26 beats per minute, p<0.001), and higher peak cardiac index (8.3±2.3 vs. 5.7±1.9 L/min/m^2^, p<0.001). These associations persisted when treating peak RVOT as a continuous variable and after age and sex adjustment. At peak exercise, patients with a high exercise RVOT gradient had both higher RV systolic pressure (78±11 vs. 66±17 mmHg, p<0.001) and lower PA systolic pressure (34±8 vs. 50±19 mmHg, p<0.001).

**Conclusions:**

Development of a systolic RV-PA pressure gradient during upright exercise is not associated with an adverse hemodynamic exercise response and may represent a normal physiologic finding in aerobically fit young people.

## Introduction

Fixed right ventricular outflow (RVOT) obstruction, such as seen with pulmonary valve stenosis, causes a systolic pressure gradient between the right ventricle (RV) and pulmonary artery (PA). Clinically significant dynamic RVOT obstruction is thought to be uncommon, but has been described in a subset of patients after cardiac surgery or after lung transplantation [1–3]. RVOT obstruction can cause right heart failure, though mild or moderate degrees of obstruction are often clinically well tolerated. Even in the face of adequate compensation, however, the presence of a pressure difference between the RV and PA during systole confounds echocardiographic estimation of PA pressures based on tricuspid regurgitation flow velocity using the simplified Bernoulli equation, which assumes RV and PA pressures are equal [4]. This is important, since echocardiographic assessment of pulmonary hemodynamics during exercise is an increasingly common and accepted part of clinical practice [5–9], particularly in specific patient subsets such as congenital heart disease and rheumatologic disease [10–15].

Dynamic left ventricular outflow tract obstruction has been extensively investigated, mainly in the context of hypertrophic cardiomyopathy [16], but this phenomenon can also occur with reduced preload [17, 18], increased contractility [19] and Takotsubo cardiomyopathy [20]. Considered anatomically, the RVOT would seem more susceptible to dynamic muscular obstruction given the presence of a circumferential, muscular, contractile infundibulum in contrast to the partly fibrous, non-contractile left ventricular outflow. Obstruction to RV ejection might be expected to impede flow from the RV to PA and cause right heart failure: decreased cardiac output and increased right heart filling pressures.

We recently presented data suggesting that a substantial number of patients without a resting RV-to-PA gradient develop a substantial pressure gradient across the RVOT during upright exercise despite an absence of specific types of structural or congenital heart disease [21]. Whether such dynamic pressure gradients are associated with disadvantageous hemodynamics, such as elevated right heart filling pressure or reduced cardiac output, are unknown. The current study presents hemodynamic and metabolic characteristics corresponding to the development of an RV-to-PA pressure gradient during exercise.

## Methods

### Study sample and design

We studied consecutive patients with unexplained exertional intolerance referred to the Dyspnea Clinic at Brigham and Women’s Hospital who underwent resting supine right heart catheterization followed by upright invasive symptom-limited cardiopulmonary exercise testing between May 2012 and May 2015. We excluded patients without RV pressure tracings during exercise (i.e., alternative catheter used or RV port located in right atrium [RA], n = 296) and those with a supine resting RVOT gradient >10mmHg (n = 16, 6 with known pulmonary valve stenosis). The Partners Human Research Committee approved this retrospective study and waived the requirement for informed consent (protocol #2011P000272).

### Right heart catheterization and exercise

Testing was performed as previously described [22]. A flow-directed, balloon-tipped, 4-port pacing PA catheter (Swan-Ganz Pacing-TD Catheter, Edwards Lifesciences, Irvine, CA, USA) was positioned into a branch PA and a radial artery line was also placed. Systemic arterial, RA, RV, PA, and PA wedge (PAWP) pressures were measured with a hemodynamic monitoring system (Xper Cardio Physiomonitoring System, Philips, Andover, MA, USA) calibrated before each study. Pressure measurements were taken at the end of a passive exhalation; when respirophasic variation persisted despite attempted passive exhalation, the electronic average over 3 respiratory cycles was used. The pressure transducer was leveled 5 cm below the axillary fold in the mid axillary line. We repeated manual measurement of all hemodynamic tracings in a subset (n = 17) for quality control. For PA systolic pressure at rest and at peak exercise, r = 0.97 (bias = 2.4±3.0 mmHg) and r = 0.98 (bias = -0.4±3.9 mmHg), respectively. For RV systolic pressure, r = 0.97 (bias = -0.1±2.5 mmHg) and r = 0.96 (bias = -2.4±4.1 mmHg) at rest and peak exercise, respectively.

All exercise tests were performed on an upright cycle ergometer with the patient breathing room air. After 2 minutes of rest followed 3 minutes of unloaded cycling at 55–65 rpm, work rate was then continuously increased until limited by symptoms. Breath-by-breath pulmonary gas exchange and minute ventilation (V_E_) were measured using a commercially available metabolic cart (Ultima CPX, MGC Diagnostics, St. Paul, MN, USA). V_E_, inspired and expired O_2_ and CO_2_ concentrations, heart rate, radial arterial pressure, RA pressure, RV pressure, and PA pressure were measured continuously; PAWP was obtained at rest and once each minute of exercise. RVOT gradient was calculated supine, upright at rest and upright at peak exercise as the difference between RV and PA systolic pressures, measured simultaneously from the respective catheter ports.

### Statistical analysis

Categorical data are expressed as number with percentages, while continuous variables are reported as mean±standard deviation or median [25^th^-75^th^ percentile] as appropriate for distribution. The cohort was divided into 2 groups using a cutoff at the 90^th^ percentile of RVOT gradient at peak exercise (≥33mmHg). Receiver-operating characteristic (ROC) curves were constructed to determine the diagnostic accuracy of systolic RV versus systolic PA pressure to identify an abnormal PA pressures response, defined as peak mean PA pressure (mPAP) >30mmHg [23, 24]. Optimal cut-points were identified using the Youden index. We repeated this analysis using a more comprehensive definition of abnormal pulmonary vascular response, peak mPAP>30mmHg and peak PVR>120 dynes·sec·cm^-5^. Predicted peak VO_2_ was estimated using published equations[25]. Continuous variables were compared between groups with the Student’s unpaired t-test for normally distributed variables and Mann-Whitney U test for non-normally distributed variables. Categorical variables were compared between groups using Fisher’s exact test. Pearson correlation analysis was performed to determine correlation between peak RVOT gradient and clinical and physiological variables. Multivariable linear regression, adjusting for age and sex, was used to identify independent predictors of development of increased RVOT gradient. We used linear regression using stepwise selection (p-value <0.10 was set for both entry to and retention in the model) to identify key clinical, demographic, or resting hemodynamic variables predictive of peak RVOT gradient. This procedure was repeated with the addition of peak exercise response variables using the same model building approach. The variables considered for inclusion in these models are listed in S1 Table.

Statistical analyses were performed using SPSS Statistics 23.0 (IBM, Chicago, IL, USA), SAS 9.3 (SAS Institute, Cary, NC, USA) and GraphPad Prism version 5.01 for Windows (GraphPad Software, La Jolla, CA, USA). A 2-tailed P<0.05 was used as the criterion for statistical significance.

## Results

### Demographics and clinical characteristics

The characteristics of the study sample are summarized in Table 1. There were 294 patients included in the analysis, 49% male with mean age of 59.7±15.5 years. Hypertension (49%), dyslipidemia (43%) and obesity (37%, body mass index ≥30 kg/m^2^) were common. Average peak VO_2_ was 17.1±7.8 mL/kg/min, or 74.8±23.3% predicted. Peak respiratory exchange ratio averaged 1.13, consistent with maximal exercise effort.

**Table 1:** Demographic and clinical characteristics of the study sample.

	Total n = 294	RVOT gradient at peak, stratified by:		*p* value	adjusted *p* value*
		< 33 mmHg n = 261	≥ 33 mmHg n = 33		
**Demographics and anthropometrics**					
Male sex	144 (49)	121 (46)	23 (70)	**0.01**	-
Age [years]	59.7±15.5	61.8±13.9	43.6±17.7	**<0.001**	-
Height [cm]	171.3±9.9	170.5±9.8	176.9±9.5	**<0.001**	0.29
Weight [kg]	85.1±21.1	85.6±21.7	81.1±16.0	0.26	0.06
BSA [m^2^]	1.97±0.25	1.96±0.26	1.98±0.21	0.79	0.16
BMI [kg/m^2^]	29.0±6.5	29.3±6.6	25.9±4.8	**0.004**	**0.02**
**Clinical characteristics**					
Hemoglobin [g/dL]	13.8±1.9	13.6±1.8	15.0±1.5	**<0.001**	0.05
FEV_1_ [% predicted]	82.2±21.8	80.8±22.0	93.2±17.6	**0.002**	0.07
FVC [% predicted]	83.3±20.3	81.9±20.2	94.3±18.1	**0.001**	0.09
FEV_1_ / FVC	0.77±0.1	0.76±0.1	0.8±0.08	**0.04**	0.54
Peak Work rate [W]	108±83	99±80	179±71	**<0.001**	0.21
Peak RER	1.13±0.13	1.12±0.13	1.2±0.09	**0.001**	0.14
Peak VO_2_ [% predicted]	74.8±23.3	72.6±20.7	92.4±33.8	**0.002**	**<0.001**
Peak VO_2_ [ml/kg/min]	17.1±7.8	15.8±6.4	27.2±9.8	**<0.001**	**<0.001**
**Cardiovascular risk factors and history**					
Hypertension	143 (49)	136 (52)	7 (21)	**0.001**	0.22
Dyslipidemia	125 (43)	117 (45)	8 (24)	**0.02**	0.68
Diabetes mellitus	42 (14)	41 (16)	1 (3)	0.06	0.25
Current tobacco use	3 (1)	3 (1)	0	1.00	1.00
CABG	21 (7)	21 (8)	0	0.15	1.00
PCI	33 (11)	33 (13)	0	**0.04**	1.00
Valvular disease	30 (10)	28 (11)	2 (6)	0.55	0.82
**Medication**					
Acetylsalicylic acid	95 (32)	91 (35)	4 (12)	**0.01**	0.21
Diuretic	89 (30)	85 (33)	4 (12)	**0.02**	0.44
Beta blocker	88 (30)	84 (32)	4 (12)	**0.02**	0.54
ACE inhibitor or ARB	59 (20)	57 (22)	2 (6)	**0.03**	0.12
Calcium channel blocker	54 (18)	51 (20)	3 (9)	0.14	0.48
Diagnosis**					
Exercise HFpEF	40 (14)	39 (15)	1 (3)	0.06	0.17
Exercise pulmonary hypertension	76 (26)	75 (29)	1 (3)	**<0.01**	0.06
Isolated low venous filling pressures	59 (20)	44 (17)	15 (45)	**<0.01**	0.09
Impaired peripheral oxygen extraction	35 (12)	31 (12)	4 (12)	1.0	0.60

Demographic and clinical characteristics for the overall study cohort and stratified by the 90^th^ percentile of peak right ventricular outflow tract (RVOT) gradient, 33 mmHg. Data are presented as mean ± SD or n (%). Data on age, sex, and peak VO_2_ for those with and without an RVOT gradient, as well as BMI and the prevalence of hypertension, dyslipidemia, CABG, PCI and beta-blocker use in the overall cohort have been previously published.[21]

*Multivariable logistic regression, adjusted for age and sex.

**Primary diagnosis based on invasive cardiopulmonary exercise test findings in the context of other clinical data. The most common primary hemodynamic diagnoses are provided; less frequent diagnoses, some of which may exist in conjunction with the primary diagnoses listed, are not presented including a pulmonary mechanical limit, chronotropic incompetence, heart failure with reduced ejection fraction, hyperventilation, systemic hypoxemia, and anemia. Likewise omitted are patients with mixed disease (e.g., peak pulmonary capillary wedge pressure >20mmHg and also peak pulmonary vascular resistance >160 dynes.s.cm^-5^). Isolated low venous filling pressure was defined as peak right atrial pressure <6mmHg in the absence of another listed diagnosis. Other diagnoses are defined as described elsewhere [22].

ACE—angiotensin-converting enzyme; ARB—angiotensin receptor blocker; BMI—body mass index; BSA—body surface area; CABG—coronary artery bypass graft; FEV_1_ —forced expiratory volume in 1 second; FVC—forced vital capacity; HFpEF—heart failure with preserved ejection fraction; PCI—percutaneous coronary intervention; RER—respiratory exchange rate.

### Dynamic RVOT gradient with upright posture and during exercise

**Fig 1:**
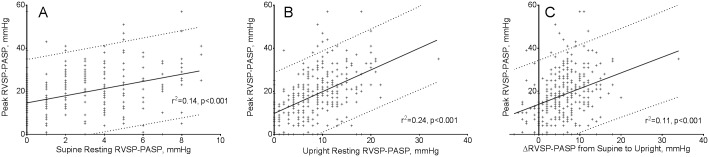
Relationships between supine and upright resting right ventricular outflow tract (RVOT) gradient and peak exercise RVOT gradient. Scatterplots of peak RVOT pressure gradient (i.e., RVSP-PASP) versus supine resting RVOT gradient (Panel A), upright resting RVOT gradient (Panel B), and the change in RVOT pressure gradient between from supine to upright positions at rest (Panel C). In the small subset of cases where resting RVSP-PASP was negative, the gradient was set to 0 mmHg. PASP—pulmonary artery systolic pressure; RVSP—right ventricular systolic pressure.

Supine resting RVOT gradient was negligible (mean 1.6±3.6 mmHg, median 2 [-1-4]; 90^th^ percentile 6 mmHg), but there was on average a modest systolic gradient between the RV and PA at rest while upright (mean 8.8±5.5 mmHg, median 9 [5–12]; 90^th^ percentile 16 mmHg). At peak exercise, the average RVOT gradient was 18.7±11.2 mmHg (median 18 [11–25]; 90^th^ percentile 33 mmHg). Peak exercise RVOT gradient was only modestly correlated with supine resting RVOT gradient (r^2^ = 0.14, p<0.001; Fig 1A), upright resting RVOT gradient (r^2^ = 0.24, p<0.001; Fig 1B), and change in resting RVOT gradient from the supine to upright position (r^2^ = 0.11, p<0.001; Fig 1C). Neither supine RVOT gradient nor change in gradient with position was independently associated with peak exercise gradient after adjustment for upright resting gradient.

Among patients with a peak RVOT gradient at or above the 90^th^ percentile (≥33 mmHg, referred to as a high RVOT gradient, n = 33), the mean gradient was 39.1±6.7 mmHg at peak exercise, while it averaged 16.1±8.7 mmHg among the other 261 patients (Table 1). Patients with a high RVOT gradient were younger (43.6±17.7 vs. 61.8±13.9 years, p<0.001), more likely to be male (70% vs. 46%, p = 0.01) and had lower body mass index (25.9±4.8 vs. 29.3±6.6 kg/m^2^, p = 0.004) than those with peak RVOT gradient <33 mmHg. To provide a better understanding of differences in baseline characteristics independent of the important differences in sex and age, we used linear regression to adjust for these variables. While those with high RVOT gradients were also taller with higher forced expiratory volume in 1 second and hemoglobin concentration, had higher peak RER, were less likely to have hypertension or diabetes mellitus, and less likely to be taking cardiovascular medications, these associations appeared to be related to the different age and sex distribution between groups (Table 1, right column).

### Resting hemodynamics

**Fig 2:**
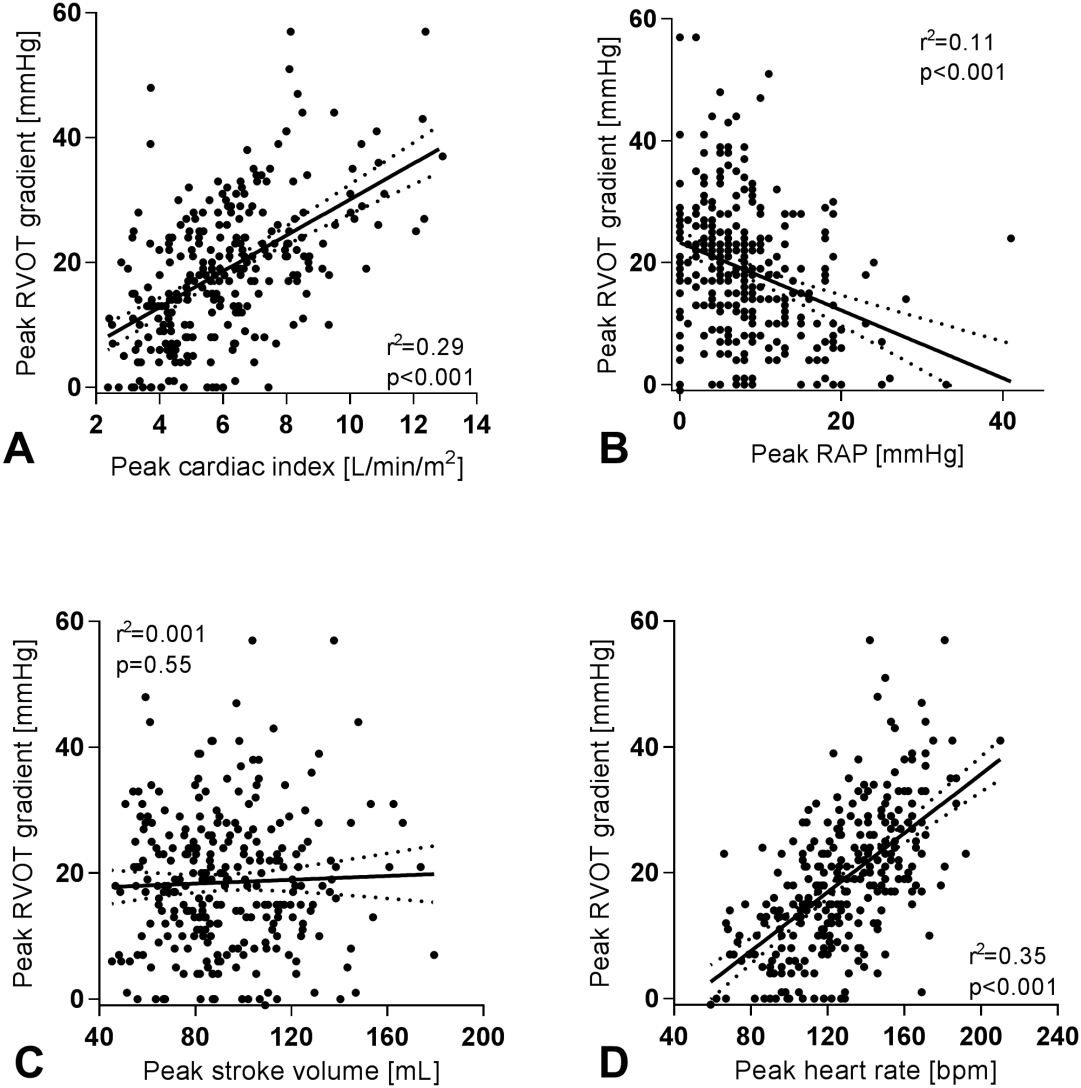
Association between selected exercise hemodynamic variables and right ventricular outflow tract (RVOT) systolic pressure gradient at peak exercise. Scatterplots showing the relationship of various peak exercise hemodynamic variables with RVOT systolic pressure gradient at peak exercise. The best-fit linear regression line is plotted with 95% confidence intervals. Panel A—Peak cardiac index versus peak RVOT gradient; Panel B—Peak RAP versus peak RVOT gradient; Panel C—Peak stroke volume versus peak RVOT gradient; Panel D—Peak heart rate versus peak RVOT gradient. RAP—right atrial pressure; RVOT—right ventricular outflow tract.

Hemodynamic data at upright rest and peak exercise are presented in Table 2. Patients with a high exercise RVOT gradient had higher resting cardiac index (3.1±0.9 vs. 2.5±0.8 L/min/m^2^, p<0.001) in the context of higher stroke volume (84±36 vs. 68±24 mL, p<0.001), and similar heart rate (p = 0.48)(Fig 2). The higher cardiac index and stroke volume were accounted for by the underlying differences in age and sex (linear regression adjusting for age and sex presented in the right most column of Table 2). Resting right atrial pressure was lower in those who developed high exercise RVOT gradient (median 0 [IQR 0–4] vs. 3 [IQR 1–6] mmHg), as was PAWP (median 4 [IQR 3–8] vs. 8 [IQR 5–11] mmHg) (p<0.001 for both). Resting pulmonary pressure was also lower at rest (mPAP 12.3±3.6 vs. 18.0±7.4 mmHg, p<0.001), due to both lower PAWP and lower pulmonary vascular resistance (100±40 vs. 172±98 dynes∙s∙cm^-5^, p<0.001). There was no statistically significant difference between groups in resting systolic RV pressure (p = 0.39).

**Table 2:** Association between resting and exercise hemodynamic variables a with peak RVOT gradient during upright cycle ergometry.

	Total n = 294	Peak RVOT gradient, stratified			Peak RVOT gradient, continuous		
		< 33 mmHg n = 261	≥ 33 mmHg n = 33	*p* value	Univariate		Multivariate*
					β	*p* value	*p* value
***Resting hemodynamics*, *upright***							
Systolic BP [mmHg]	146±23	146±24	145±20	0.8	0.02	0.59	**0.007**
Diastolic BP [mmHg]	79±13	78±13	81±11	0.18	0.16	**0.002**	0.09
Stroke volume [mL]	69.2±26.1	67.6±24.4	84.1±35.5	**0.001**	0.04	0.09	0.7
Heart rate [bpm]	75±14	75±13	77±20	0.48	0.08	0.09	0.1
Cardiac output [L/min]	5.0±1.7	4.9±1.6	6.2±1.8	**<0.001**	1.09	**0.005**	0.97
Cardiac index [L/min/m^2^]	2.6±0.8	2.5±0.8	3.1±0.9	**<0.001**	2.24	**0.004**	0.54
Right atrial pressure [mmHg]	3 [0–6]	3 [1–6]	0 [0–4]	**<0.001**	-1.09	**<0.001**	**<0.001**
PCW pressure [mmHg]	7 [4–11]	8 [5–11]	4 [3–8]	**<0.001**	-0.82	**<0.001**	**<0.001**
Mean PA pressure [mmHg]	17.3±7.3	18±7.4	12.3±3.6	**<0.001**	-0.64	**<0.001**	**<0.001**
Systolic PA pressure [mmHg]	25±11.3	25.9±11.6	18.0±5.2	**<0.001**	-0.38	**<0.001**	**<0.001**
Systolic RV pressure [mmHg]	33.8±10.8	34±11.1	32.2±8.1	0.39	-0.16	**0.009**	0.18
PVR [dynes.s.cm^-5^]	164±96	172±98	100±40	**<0.001**	-0.03	**<0.001**	**0.03**
RVOT gradient [mmHg]	8.8±5.5	8.1±5.1	14.3±6.2	**<0.001**	1.01	**<0.001**	**<0.001**
***Peak exercise hemodynamics***							
Systolic BP [mmHg]	180±38	179±37	184±45	0.48	0.06	**0.001**	**0.003**
Diastolic BP [mmHg]	82±18	82±17	86±20	0.17	0.14	**<0.001**	0.33
Stroke volume [mL]	92.6±25.8	91.4±25.7	103.7±24.5	**0.02**	0.08	**0.003**	0.99
Heart rate [bpm]	128±28	124±26	159±19	**<0.001**	0.24	**<0.001**	**<0.001**
Cardiac output [L/min]	11.8±4.3	11.3±3.9	16.6±4.4	**<0.001**	1.29	**<0.001**	**<0.001**
Cardiac index [L/min/m^2^]	6±2	5.7±1.9	8.3±2.3	**<0.001**	2.91	**<0.001**	**<0.001**
Right atrial pressure [mmHg]	7 [4–11]	8 [4–12]	5 [3–7]	**<0.001**	-0.57	**<0.001**	**<0.001**
PCW pressure [mmHg]	14 [10–20]	14 [10–21]	12 [10–15]	**0.04**	-0.37	**<0.001**	**0.001**
Mean PA pressure [mmHg]	32.5±11.9	33.3±12.2	26.1±5.9	**<0.001**	-0.34	**<0.001**	**<0.001**
Systolic PA pressure [mmHg]	48.7±18.1	50±18.7	38.8±7.6	**<0.001**	-0.25	**<0.001**	**<0.001**
Systolic RV pressure [mmHg]	67.3±17.1	66±17.4	77.9±10.7	**<0.001**	0.15	**<0.001**	**<0.001**
PVR [dynes.s.cm^-5^]	134±98	140±101	78±41	**<0.001**	-0.04	**<0.001**	**<0.001**
RVOT gradient [mmHg]	18.7±11.2	16.1±8.7	39.1±6.7	**<0.001**	-	-	-
***Change*, *rest to peak exercise***							
Stroke volume [mL]	23.3±24.9	23.7±23.9	19.5±33.2	<0.001	0.04	0.18	0.72
Heart rate [bpm]	53±26	49±24	82±20	0.41	0.26	**<0.001**	**<0.001**
Cardiac output [L/min]	6.8±3.8	6.4±3.5	10.4±4.2	**<0.001**	1.45	**<0.001**	**<0.001**
Cardiac index [L/min/m2]	3.4±1.8	3.2±1.7	5.2±2.2	**<0.001**	3.1	**<0.001**	**<0.001**
Right atrial pressure [mmHg]	4 [1–7]	4 [1–7]	3 [0–5]	0.13	-0.47	**0.001**	**0.003**
PCW pressure [mmHg]	7 [4–10]	7 [3–11]	7 [4–10]	0.51	-0.02	0.87	0.63
Mean PA pressure [mmHg]	15.2±7.8	15.4±8.1	13.8±4.7	0.11	-0.23	**0.006**	**0.002**
Systolic PA pressure [mmHg]	23.7±11.0	24.1±11.4	20.8±7.3	**0.03**	-0.26	**<0.001**	**<0.001**
Systolic RV pressure [mmHg]	33.5±12.6	32±11.9	45.6±11.4	**<0.001**	0.4	**<0.001**	**<0.001**
RVOT gradient [mmHg]	9.8±9.9	7.9±8.2	24.8±9.4	**<0.001**	0.98	**<0.001**	**<0.001**

Hemodynamic variables at rest, peak exercise, and change between rest and peak exercise are presented for the whole cohort and stratified according to the 90^th^ percentile of peak RVOT gradient, 33mmHg. Data are presented as mean ± SD or median [25th– 75th percentile] as appropriate for distribution. Univariate linear regression coefficients are presented. The upright resting and peak RVOT gradients for the overall cohort (8.8 ± 5.5 mm Hg and 18.7 ± 11.2 mm Hg, respectively) have been published previously [21].

*Multivariable linear regression, adjusted for age and sex.

BP—blood pressure; PA—pulmonary artery; PCW—pulmonary capillary wedge pressure; PVR—pulmonary vascular resistance; RV—right ventricle; RVOT—right ventricular outflow tract.

### Hemodynamics at peak exercise

The hemodynamic pattern at peak exercise was largely, though not entirely, similar to the resting findings. Those with high exercise RVOT gradient had higher peak cardiac index (8.3±2.3 vs. 5.7±1.9 L/min/m^2^, p<0.001), in the context of both higher peak heart rate (159±19 vs. 124±26 bpm, p = 0.001) and stroke volume (104±25 vs. 91±26 mL/beat, p<0.001). The higher stroke volume was related to differences in age and sex, while the relationship between higher heart rate and higher RVOT gradient persisted after adjustment for these covariates (Table 2). Furthermore, both RA pressure and PAWP remained significantly lower during exercise in the high RVOT gradient group, as did pulmonary vascular resistance. Importantly, mPAP remained lower in the high RVOT gradient group at peak exercise and there was no difference between those who did and did not develop a high RVOT gradient in the change in mPAP during exercise (+13.8±4.7 vs. +15.4±8.1 mmHg, p = 0.11). RV systolic pressure increased by 45.6±11.4 mmHg in patients with high RVOT gradient, but only 32.0±11.9 mmHg for the patients with RVOT gradient <33mmHg at peak exercise (p<0.001).

### Multivariable predictors of higher RVOT gradient

Multivariable linear regression identified several resting/baseline predictors of peak RVOT gradient (Table 3, top section). The predictors which correlated most strongly with increased peak exercise RVOT gradient included lower resting mPAP, younger age, and male sex. Other variables associated with higher gradient were higher resting systolic blood pressure, not taking a beta-blocker medication, higher forced vital capacity, and higher resting heart rate.

**Table 3:** Multivariable predictors of peak RVOT gradient during upright cycle ergometry.

**Baseline/Resting Variables Alone**			
Variable	β coefficient	P value	Partial r^2^
Mean PAP, upright	-0.48	<.0001	0.102
Age	-0.19	<.0001	0.086
Sex, male	4.69	<.0001	0.071
SBP, rest	0.08	0.0004	0.046
Taking beta-blocker	-4.15	0.0008	0.042
FVC, % predicted	0.08	0.01	0.025
Heart rate, rest	0.07	0.06	0.013
**Baseline/Resting Plus Peak Exercise Variables**			
Variable	β coefficient	P value	Partial r^2^
Peak HR	0.17	<.0001	0.164
mPAP, peak	-0.37	<.0001	0.153
Cardiac output, peak	0.46	0.003	0.037
SBP, rest	0.09	0.003	0.036
PAWP, peak	0.31	0.003	0.036
[Hgb]	0.81	0.009	0.028
RAP, peak	-0.28	0.02	0.023
DBP, rest	-0.12	0.03	0.018

Top: A multivariable model of baseline predictors of the magnitude of RVOT pressure gradient with exercise, continuous variable per mmHg. Variable selection was performed in a stepwise manner, with a p-value <0.1 required for both entry and retention in the model.

Use of a simpler forward selection approach with p for entry <0.1 resulted in inclusion of the same variables, but with the addition of hemoglobin concentration in the model (for hemoglobin concentration, final p = 0.16 and partial r^2^ = 0.007).

Bottom: A multivariable model of independent correlates of development of an RVOT pressure gradient with exercise, including both baseline/resting data and peak exercise variables. The same approach to model selection was applied as for the resting model. Use of a simpler forward selection approach with p for entry <0.1 produced the same result.

Variables considered for inclusion in both models are listed in S1 Table. Type II partial Pearson correlation coefficients are also presented.

BPM—beats per minute; FVC—forced vital capacity; [Hgb]—hemoglobin concentration; PAWP—pulmonary artery wedge pressure; mPAP—mean pulmonary artery pressure; PVR—pulmonary vascular resistance; RAP—right atrial pressure; S/DBP—systolic/diastolic blood pressure.

When peak hemodynamic and other exercise variables were considered in addition to the resting/baseline data, model performance improved and the strongest correlates of higher peak RVOT gradient were higher peak heart rate and lower peak mPAP (Table 3, bottom section). Several other variables, including peak cardiac output and right atrial pressure, were only weakly predictive of peak RVOT gradient after multivariable adjustment. Higher hemoglobin concentration as well as both higher systolic and lower resting diastolic resting blood pressure, were also associated with higher peak RVOT gradient, albeit quite modestly. As importantly, when exercise variables were considered neither age nor sex remained an independent predictor of RVOT gradient.

### Sensitivity analyses

There was no apparent heterogeneity among demographic, hemodynamic or diagnostic subgroups in terms of the positive relationship between higher RVOT gradient at peak exercise and greater peak VO_2_. These analyses included stratification by sex, age (by median value), peak RAP (< vs. ≥7mmHg), peak PAWP (< vs. ≥21mmHg), peak arterial-venous O_2_ content difference (< vs ≥90% of resting hemoglobin concentration), peak heart rate (< vs ≥85% predicted peak heart rate) and body mass index (< vs. ≥26 kg/m^2^). We further assessed for 2-way interaction between peak RVOT gradient and each of these variables, as both continuous and categorical variables. There were no statistically significant 2-way interactions. That is, there was no indication that higher RVOT gradient may be related to worse aerobic capacity in any subset.

### Relationship between pulmonary artery and right ventricular pressure response

**Fig 3:**
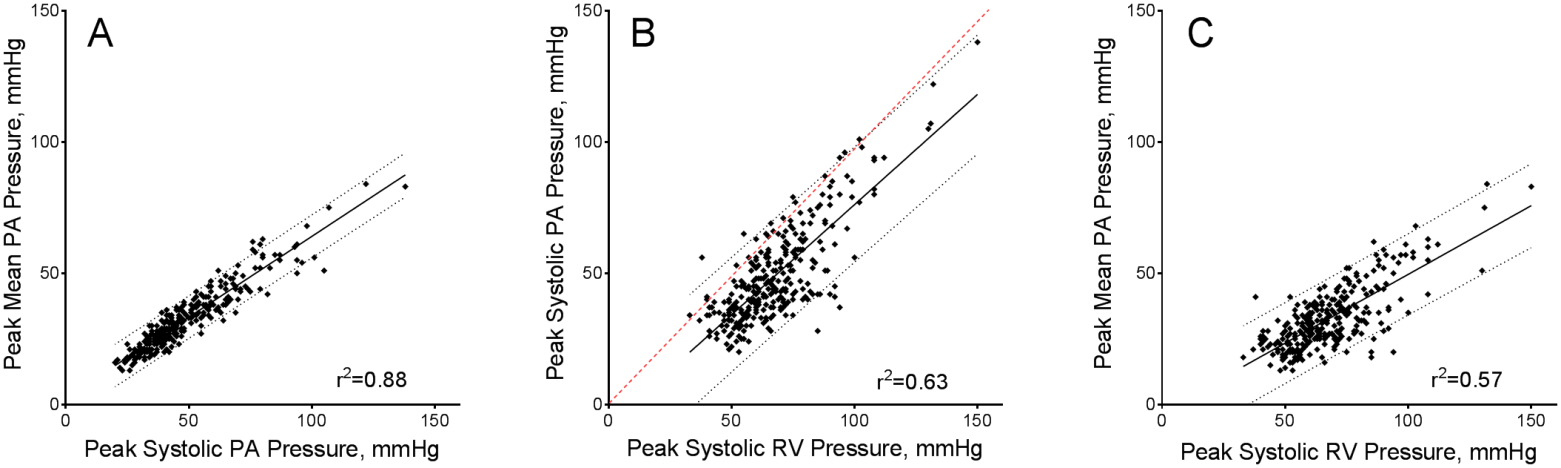
Relationships between peak right ventricular systolic and pulmonary artery systolic and mean pressures at peak exercise. (Panel A) Scatterplot showing the close correspondence of invasively measured peak exercise systolic pulmonary artery (PA) pressure and mean PA pressure. (Panel B) Scatterplot of invasively measured peak exercise systolic right ventricular (RV) pressure against peak exercise systolic PA pressure. Peak RV systolic pressure is systematically higher than peak PA systolic pressure. Also, the correlation between RV and PA systolic pressures is less robust than would be expected. (Panel C) As a result of the systematic but variable RV-to-PA systolic pressure gradient, the relationship between peak systolic RV pressure and peak mean PA pressure is only moderately strong. For all panels, the solid black line represents the best-fit regression line with dotted lines representing 95% prediction limits. The dashed red line in panel B signifies identity (x = y). PA- pulmonary artery; RV- right ventricle.

As noted in our prior report [21], there was a close relationship between invasively measured systolic and mean PA pressure at peak exercise (r^2^ = 0.88, Fig 3A). Due to the presence of a variable pressure gradient across the RVOT during exercise, RV systolic pressure systematically overestimated systolic PA pressure or mean PA pressure (r^2^ = 0.63 and r^2^ = 0.57, respectively; Fig 3B and 3C). Half (n = 147) of the patients demonstrated an abnormal pulmonary pressure response during exercise, defined as peak mPAP >30 mmHg. Almost all patients with a hypertensive pulmonary pressure response also had elevated RV systolic pressure (99%, n = 145/147 >50mmHg). However, many patients with normal exercise PA pressure also had elevated RV systolic pressure at peak exercise (>50mmHg in 75%, >60mmHg in 37%, and >70mmHg in 15.6%).

**Fig 4:**
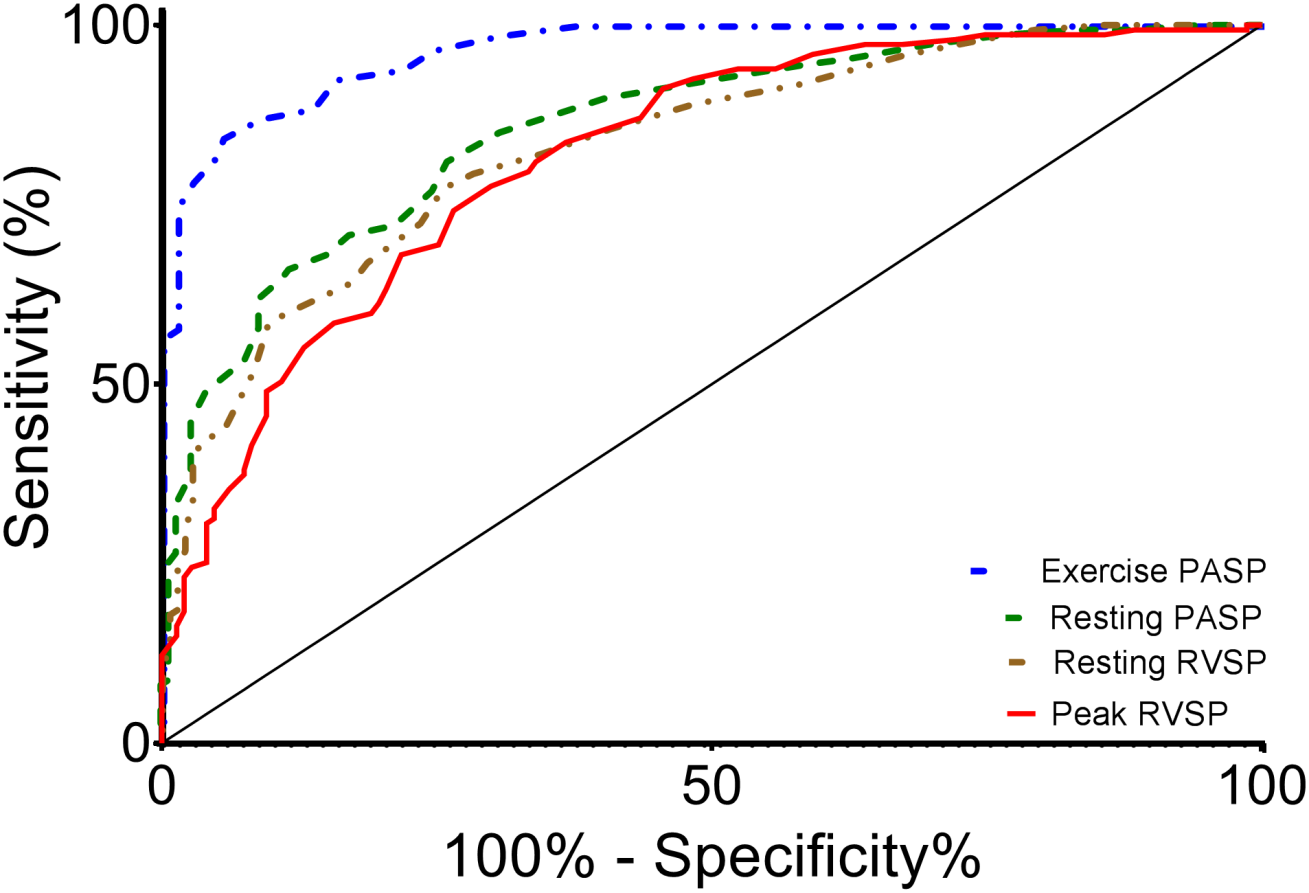
Receiver operating characteristic curve analysis of resting and peak exercise right heart pressures to identify peak exercise mean pulmonary artery pressure >30 mmHg. Peak exercise PA systolic pressure is able dependably to identify patients with abnormally high mean PA pressure at peak exercise (AUC 0.97, blue dotted-dashed line). Peak exercise RVSP is less well able to discriminate between normal and elevated exercise mean PA pressure (AUC 0.82, red solid line). Resting supine right heart catheterization RV systolic pressure (AUC 0.83, brown dashed double-dotted line) and PA systolic pressures (AUC 0.86, green dashed line) each provided similar or slightly better discrimination between normal and elevated exercise PA pressure. PA—pulmonary artery; PASP—pulmonary artery systolic pressure; RV—right ventricle; RVSP—right ventricle systolic pressure.

Receiver operating characteristic analysis identified optimal cutoff points for RV systolic pressure and PA systolic pressure for an abnormal pulmonary pressure response during exercise (mPAP>30 mmHg). PA systolic pressure >43 mmHg provided sensitivity and specificity of 93% and 84%, respectively, to identify abnormal mPAP response (AUC = 0.97, 95% CI 0.95–0.98) (Fig 4, blue dashed-dotted line). Systolic RV pressure was less predictive; a value >61 mmHg had sensitivity and specificity of 84% and 63%, respectively (AUC = 0.82, 95% CI 0.77–0.87)(Fig 4, red solid line). To provide context, both resting supine right heart catheterization PA systolic and RV systolic pressures (Fig 4, AUC = 0.86 and 0.83, respectively) were at least as predictive of abnormally elevated peak exercise mPAP. Both PA and RV systolic pressure were modestly less predictive when a more comprehensive definition of abnormal pulmonary vascular response was used (peak mPAP>30mmHg and peak PVR>120 dynes·sec·cm^-5^, n = 82); for PA systolic pressure and RV systolic pressure, AUC were 0.92 and 0.76, respectively.

## Discussion

We recently reported that a subset of patients referred for invasive evaluation of effort intolerance develop a pressure gradient across the RVOT during upright exercise, despite the absence of a resting gradient or known anatomic reason for dynamic obstruction [21]. That brief report, from the same cohort included in the present analysis, noted that the presence of an RVOT gradient was more common in younger, male patients and was associated with higher peak VO_2_. The current paper further describes clinical, hemodynamic and metabolic features associated with the development of such a gradient. Most importantly, the existence of an exercise RVOT gradient was not accompanied by clinical or hemodynamic features of right heart failure. In fact, the converse was seen: a high RVOT gradient during exercise was associated with low biventricular filling pressures, more robust cardiac output response, and higher peak VO_2_. These findings suggest that a pressure difference between the RV and PA may represent a newly appreciated but possibly normal hemodynamic response to exercise in well-conditioned young people. Confirmation of that hypothesis will require validation in independent cohorts of healthy people.

We can only speculate as to a possible functional reason for the finding described based on the available data. The RVOT, or infundibulum, is anatomically and embryologically distinct from the main body of the RV, as described by Arthur Keith in 1924 [26]. Sir Keith also hypothesized a function for the RVOT in normal humans: “the safeguarding of the capillary system of the lungs from high and prolonged accessions of blood pressure”, as would be seen during periods of exertion or stress [26]. This would parallel the function of the bulbus cordis in lower vertebrates. Alternatively, elevated RV systolic pressure at high levels of exercise may help preserve advantageous ventricular interaction (e.g., prevent collapse of the RV inflow, which could impede right-sided early diastolic filling) when left ventricular systolic pressure is markedly elevated. It is also possible that this finding is simply an epiphenomenon, the logical extreme consequence of left ventricular and septal contribution to RV contraction.

The RVOT has distinct contraction patterns and responses to inotropic agents [27]. Under normal conditions, the infundibulum contracts ~20–50 msec after the body of the RV and maintains contraction into diastole [28–30]. Sympathetic stimulation or norepinephrine injection can suppress this delay [31]. One might hypothesize that the infundibulum’s catecholamine response protects the pulmonary vasculature from high pressure during episodes of extreme exertion while maintaining advantageous peristaltic contraction. Keith’s early 20^th^-century hypothesis and more recent physiologic observations, therefore, are consistent with our findings that a dynamic RVOT gradient occurs more frequently in the presence of high cardiac output. On the other hand, this observation could simply reflect higher flow across a similar anatomic obstruction. RVOT anatomy (relatively long and narrow) would seem to predispose to such a response. A flow-based mechanism alone, though, could not explain the increase in gradient from supine to upright position, since cardiac output is lower in the upright compared with supine position [32]. The slow frequency response of a fluid filled catheter precludes comment on the timing of gradient during systole. Clarification of the underlying mechanism of the observed RVOT gradient will require more detailed investigation.

Whatever the underlying causes and consequences of dynamic RVOT obstruction with exercise, this finding has implications for the use of echocardiography to quantify pulmonary pressure response during exercise. The current data suggest echocardiography would substantially overestimate pulmonary artery systolic pressure in a subset of patients. This concern, though, may be more relevant when screening lower risk populations; there was a low prevalence of exercise pulmonary hypertension among patients with high RVOT gradients. Studies have shown that assessment of the pulmonary circulation during exercise can identify early or latent pulmonary vascular disease in patients at risk of developing pulmonary hypertension [10, 12, 33]. It may also help define underlying pathophysiology among patients with high-normal pulmonary artery pressure, who are at increased risk for adverse outcomes [34]. An abnormal pulmonary vascular response during exercise has furthermore been associated with decreased exercise capacity and development of resting pulmonary arterial hypertension, highlighting the clinical importance of ‘exercise-induced’ pulmonary hypertension and the possible role for noninvasive screening in high-risk patient subgroups [35–38].

Of note, while high RVOT gradients during exercise were not associated with a clear pathologic finding in terms of usual definitions of cardiovascular dysfunction (i.e., higher filling pressure, lower cardiac index, lower VO_2_), the subjects studied were referred because of exertional symptoms. In that context, it is possible that such high RVOT gradients may play a pathologic role in specific patient groups, as has been described in those recovering from cardiac surgery [1]. Patients with low venous pressure during exercise were prone to develop high RVOT gradients. Dynamic RVOT obstruction could presumably be part of the mechanism of symptoms in such patients [39], as it may in a subset of patients with hypertrophic cardiomyopathy [40]. This could help explain the divergent response of such patients to pharmacologic agents (e.g., beta-blockers). Patients for whom RVOT obstruction is playing a role may selectively benefit from these medications. These remain speculative hypotheses; we were unable to identify any subset of patients where higher RVOT gradient was associated with lower aerobic capacity. This could, however, be due to limited subgroup sample size or confounding mechanisms underlying symptoms. It will be necessary to study normal, asymptomatic individuals to better understand whether it is truly normal to develop a dynamic pressure gradient across the RVOT.

We did not perform a direct comparison of invasive measurement and echocardiographic estimation of the PA pressure, but precise invasive measurement of RV systolic pressure should be the best possible scenario for the use of Doppler tricuspid regurgitant systolic velocity to estimate PA systolic pressure. Moreover, while using mPAP>30mmHg is not a comprehensive definition of abnormal exercise pulmonary vascular response, this approach only favors RV systolic pressure, whether invasive or non-invasive. Of note, though these findings strongly imply that upright exercise echocardiographic estimation of PA systolic pressure would be non-specific, echocardiography is able to provide a more nuanced and comprehensive view of pulmonary vascular physiology including estimates of resistance [41, 42].

These results must be interpreted within the limitations intrinsic to the study design. Most fundamentally, the study sample was comprised of symptomatic patients with heterogeneous pathophysiology. This limits inference about whether the findings described may be normal, a normal variant, a beneficial physiologic response to high flow, or a novel pathophysiologic underpinning of dyspnea in a discrete set of patients. The spectrum of underlying disease is also particular to this setting, both because of institutional referral patterns and since invasive exercise testing is usually reserved for situations where non-invasive and resting invasive testing are insufficient. This would tend to enrich the study sample for patients with difficult to diagnose conditions such as disorders of oxygen extraction (e.g., mitochondrial disorders), exercise pulmonary hypertension, and isolated impairment of venous return. These biases do not threaten the fundamental validity of our observations, but do limit their generalizability. That is, the prevalence and magnitude and relevance of exercise RVOT gradients in the general population cannot be inferred from the available data. We also cannot entirely exclude the possibility that the gradient described is artifactual. One could propose, for example, there may be selective ring artifact (overshoot) for the RV port/lumen but not the PA or RA port/lumens related to the higher frequency components of RV pressure. Alternatively, one could hypothesize a local pressure effect of contracting RV muscle bundles. Artifact, however, seems unlikely to explain our findings for a number of reasons. First, an RVOT gradient was never seen during supine rest but was present during upright rest and with exercise in a subset of patients. One would expect the conditions that create the artifact to be present in at least a small number of patients in the supine position. Second, the finding was consistent; there was never a reversed gradient with PA pressure being substantially higher than RV pressure. Third, RV diastolic pressures were not affected. Fourth, prior studies using this catheter in supine animals and humans have not reported the presence of a systolic pressure difference between the RV and PA, [43, 44] while others have reported in post-operative patients a similar phenomenon (in that context, however, it was associated with reduced cardiac output) that was reversible by medical intervention [1, 44]. Sex and body size were associated with RVOT gradient severity. It is plausible that the RV port is located more distally (i.e., in the RVOT distal to any obstruction) in larger patients. This, however, would bias towards a lower mean RVOT gradient in taller patients, the converse of what was seen. Finally, it remains unknown whether such an RVOT gradient occurs during supine or semi-supine exercise. Further study is required to define whether these findings have any direct ramifications for standard clinical stress echocardiography, which is usually performed supine. If this phenomenon is limited to exercise in the upright position, however, it adds to the list of fundamental positional differences in physiologic exercise response; since most day-to-day physical work is performed while upright, this implies supine exercise testing may be suboptimal.

## Conclusion

The development of a pressure gradient between the RV and PA during upright exercise does not appear to be associated with an adverse hemodynamic profile. Further investigation is needed to determine whether the finding described is due to normal physiology or represents a specific pathophenotype among patients with unexplained effort intolerance. In either case, however, these findings raise questions about the application during exercise of echocardiographic methods commonly used to estimate systolic pulmonary artery pressure.

## Supporting information

S1 Table Variables considered for inclusion in the linear regression model. *Top*: A list of variables considered for inclusion in a multivariable model to indicate independent resting predictors of development of an RVOT pressure gradient with exercise. Variable selection was performed in a stepwise manner, with p value <0.1 required for entry and retention in the model. We selected a subset of variables among those that were highly correlated or mathematically related (e.g., resting upright systolic, mean and diastolic PA pressure; resting supine and upright systolic PA pressure; PVR, transpulmonary gradient, PAWP and cardiac output). Variables that could define resting upright RV pressure gradient were also omitted from consideration (i.e., upright right ventricular and pulmonary artery systolic pressure).
Use of a simpler forward selection approach with p for entry <0.1 resulted in inclusion of the same variables, but with the addition of hemoglobin concentration in the model (for hemoglobin concentration, final p = 0.16 and partial r^2^ = 0.007).
*Bottom*: A list of variables considered, in addition to all resting data listed, for inclusion in a multivariable model to indicate independent correlates of development of an RVOT pressure gradient with exercise. Use of a simpler forward selection approach with p for entry <0.1 resulted in the same final model.
ACE—angiotensin converting enzyme; ARB—angiotensin receptor blocker; BMI—body mass index; BSA—body surface area; CABG—coronary artery bypass graft; FEV1 —forced expiratory volume in 1 second; FVC—forced vital capacity; PCI—percutaneous coronary intervention; PAWP—pulmonary artery wedge pressure; PA—pulmonary artery; PVR—pulmonary vascular resistance.
(DOCX)
