# Supplementary material for: Hemodynamic and metabolic characteristics associated with development of a right ventricular outflow tract pressure gradient during upright exercise
Source: PLoS One. 2017 Jun 21;12(6):e0179053. doi: 10.1371/journal.pone.0179053 (PMC5479527; doi:10.1371/journal.pone.0179053)
Supplement: S1 Table — Top: A list of variables considered for inclusion in a multivariable model to indicate independent resting predictors of development of an RVOT pressure gradient with exercise. Variable selection was performed in a stepwise manner, with p value <0.1 required for entry and retention in the model. We selected a subset of variables among those that were highly correlated or mathematically related (e.g., resting upright systolic, mean and diastolic PA pressure; resting supine and upright systolic PA pressure; PVR, transpulmonary gradient, PAWP and cardiac output). Variables that could define resting upright RV pressure gradient were also omitted from consideration (i.e., upright right ventricular and pulmonary artery systolic pressure). Use of a simpler forward selection approach with p for entry <0.1 resulted in inclusion of the same variables, but with the addition of hemoglobin concentration in the model (for hemoglobin concentration, final p = 0.16 and partial r2 = 0.007). Bottom: A list of variables considered, in addition to all resting data listed, for inclusion in a multivariable model to indicate independent correlates of development of an RVOT pressure gradient with exercise. Use of a simpler forward selection approach with p for entry <0.1 resulted in the same final model. ACE—angiotensin converting enzyme; ARB—angiotensin receptor blocker; BMI—body mass index; BSA—body surface area; CABG—coronary artery bypass graft; FEV1 —forced expiratory volume in 1 second; FVC—forced vital capacity; PCI—percutaneous coronary intervention; PAWP—pulmonary artery wedge pressure; PA—pulmonary artery; PVR—pulmonary vascular resistance. (DOCX) [file pone.0179053.s001.docx]

Supplemental Table 1

Top: Resting variables considered for inclusion as predictors in the multivariable model.

Age

Body surface area

Sex

Height

Weight

Body mass index

Hemoglobin concentration

Heart rate

Systolic blood pressure

Diastolic blood pressure

Oxygen saturation

*Arterial blood gas variables and expired gas variables*:

pH

PaO_2_

PAaO_2_

PaCO_2_

PETCO_2_

PaETO_2_

Lactate concentration

*Comorbidities*:

History of CABG

History of PCI

History of valve disease

Diabetes

Dyslipidemia

Family history of coronary disease

Hypertension

Tobacco use

*Spirometry*:

FEV_1_:FVC

FEV_1_:FVC, % predicted

FEV_1_, % predicted

FVC, % predicted

*Resting supine hemodynamic data*:

Cardiac index

PA oxygen saturation

PAWP

PVR

Right atrial pressure

Stroke Volume

Systolic PA pressure

*Medications*:

ACE inhibitors/ARB

Aspirin

Beta-blockers

Calcium channel blockers

Digoxin

Diuretics

Insulin

Oral hypoglycemic drugs

*Upright resting hemodynamic data*:

Cardiac output

PAWP

PVR

Right atrial pressure

Stroke Volume

Diastolic PA pressure

Mean PA pressure

Bottom: Additional peak exercise variables considered for inclusion in a second model.

*Peak arterial blood gas variables and expired gas variables*:

pH

PaO_2_

PAaO_2_

PaCO_2_

PETCO_2_

PaETO_2_

Lactate concentration

*Upright peak hemodynamic data*:

Cardiac output

PAWP

PVR

Right atrial pressure

Stroke Volume

Diastolic PA pressure

Mean PA pressure

Heart rate

Systolic blood pressure

Diastolic blood pressure
